# Effect of Conformation of Sugar Beet Pectin on the Interfacial and Emulsifying Properties

**DOI:** 10.3390/foods11020214

**Published:** 2022-01-13

**Authors:** Benjamin Bindereif, Heike Petra Karbstein, Katharina Zahn, Ulrike Sabine van der Schaaf

**Affiliations:** Chair of Food Process Engineering, Institute of Process Engineering in Life Sciences, Karlsruhe Institute of Technology, 76131 Karlsruhe, Germany; benjamin.bindereif@kit.edu (B.B.); heike.karbstein@kit.edu (H.P.K.); katharina.zahn@partner.kit.edu (K.Z.)

**Keywords:** sugar beet pectin, emulsions, pH dependency, conformation, adsorbed mass, layer thickness, quartz crystal microbalance

## Abstract

The influence of the conformation of sugar beet pectin (SBP) on the interfacial and emulsifying properties was investigated. The colloidal properties of SBP, such as zeta potential and hydrodynamic diameter, were characterized at different pH levels. Furthermore, pendant drop tensiometry and quartz crystal microgravimetry were used to study adsorption behavior (adsorbed mass and adsorption rate) and stabilizing mechanism (layer thickness and interfacial tension). A more compact conformation resulted in a faster reduction of interfacial tension, higher adsorbed mass, and a thicker adsorption layer. In addition, emulsions were prepared at varying conditions (pH 3–5) and formulations (1–30 wt% MCT oil, 0.1–2 wt% SBP), and their droplet size distributions were measured. The smallest oil droplets could be stabilized at pH 3. However, significantly more pectin was required at pH 3 compared to pH 4 or 5 to sufficiently stabilize the oil droplets. Both phenomena were attributed to the more compact conformation of SBP at pH < pK_a_: On the one hand, pectins adsorbed faster and in greater quantity, forming a thicker interfacial layer. On the other hand, they covered less interfacial area per SBP molecule. Therefore, the SBP concentration must be chosen appropriately depending on the conformation.

## 1. Introduction

Pectins are complex heterogeneous polysaccharides located in the cell walls of higher plants. Pectin’s specific structural composition is strongly influenced by its origin and extraction method [1,2,3]. Due to their varying molecular structure, pectin’s emulsifying properties differ from one pectin type to another [2,4]. Thus, depending on the pectin’s type and composition, pectins find several potential applications in the food, pharmaceutical, and cosmetic industries. For example, citrus and apple pectins are widely used to increase viscosity and act as thickening, stabilizing, and gelling agents [5,6,7]. In contrast to other pectins, sugar beet pectins (SBPs) are still not widely used in food applications, as SBPs lack gelling properties [7,8,9]. Nevertheless, SBPs are gradually gaining acceptance as emulsifying agents in emulsion-based products due to their favorable molecular composition and consequently their excellent emulsifying properties [4,10,11].

Additionally, SBP as emulsifying agents provide more advantages compared to other hydrocolloids; unlike other hydrocolloids, such as gum arabic or soybean soluble polysaccharides, one requires significantly lower concentrations of SBP in order to successfully stabilize oil droplets [12]. Since SBPs are gained from waste streams of the sugar industry, SBPs allow for the production of pectin-stabilized vegan foods and beverages with emulsifying agents of regional and sustainable origin [11,13].

Even though pectins may have different molecular features, all are composed of three major domains: homogalacturonan (HG), rhamnogalacturonan I (RG-I), and rhamnogalacturonan II (RG-II). HG is the most abundant, and it builds pectin’s “smooth region”. This linear homopolymer consists of α-(1,4)-linked D-galacturonic acid monomers, each of which can be both methyl-esterified and acetyl-esterified [14]. Depending on environmental conditions and on pectin’s degree of methyl-esterification, the carboxyl groups found in the D-galacturonic acid monomers can dissociate, thus providing a negative charge to the molecule.

SBP’s emulsifying properties are attributed to its functional groups and molecular structure. Compared to other pectins, SBPs are smaller, contain higher amounts of protein, ferulic acid, and neutral sugar side chains, and possess a higher degree of acetylation [2,4,7,14,15,16]. These structural features seem to have a positive effect on the emulsifying properties, as proposed in the literature [4,7,10,16,17,18,19,20].

Aside from the structural properties of the pectin, the environmental conditions of the continuous phase, such as pH or ionic strength, affect SBP’s colloidal properties (e.g., conformation and surface charge density) and thus their functionality as emulsifiers [12,21,22,23].

Schmidt, Schütz, and Schuchmann [24] showed that the pH value influences the colloidal properties of citrus pectins between pH 2 to 4 and therefore the adsorption kinetics. Lower pH and higher ionic strengths lead to a more compact pectin size, a reduced molecule charge density, and a faster droplet adsorption. Zhao et al. [25] also demonstrated that citrus pectins with a more compact conformation at pH 2 resulted in a greater reduction of the interfacial tension than more unfolded molecules at pH 7. This was attributed to the fact that more pectins can adsorb to the interface due to the compact structure and less electrostatic repulsion. In addition, it was shown that as a result of reduced intermolecular repulsions, citrus pectins form a dense and more compact film, with an enlarged layer thickness at the interface [24,25]. Since the structure of SBP is significantly different from that of citrus pectins, i.e., the neutral sugar content is much higher, it is necessary to investigate whether this also applies to SBP.

The objective of this study was to investigate the emulsifying and emulsion stabilizing properties of SBP at varying formulations and environmental conditions. The authors expected that SBP, with an uncharged and compact conformation (at pH values below the pK_a_ of galacturonic acid of ~3.5), should be able to adsorb onto an oil-water interface faster and in greater numbers than more extended and charged pectins [26,27]. This might improve the emulsifying properties, as the droplets can be stabilized faster after droplet break-up, which reduces coalescence. Moreover, this could lead to the formation of thick interfacial layers, thus providing effective steric stabilization of oil droplets [22,28].

## 2. Materials and Methods

### 2.1. Materials

Sugar beet pectin was provided by Herbstreith & Fox KG. (Neuenbürg, Germany). The main molecular characteristics are protein content: 4.1% ± 0.2%; molecular weight: 104 kDa ± 5 kDa; galacturonic acid: 66.1% ± 1.8%; degree of methylation: 52.1% ± 1.7%; degree of acetylation: 23.1% ± 0.5%; and trans-ferulic acid: 706 mg/100g ± 21 mg/100 g [18].

Medium chain triglyceride oil (MCT oil) was supplied by IOI Oleo GmbH (Hamburg, Germany). The MCT oil was composed of 60% C_8_ and 40% C_10_ chains and had a density of 0.95 kg/L at room temperature, according to supplier information. 1-Dodecanthiol (≥98%), hydrogen peroxide (30%), and sulfuric acid (98%) were purchased from Merck KGaA (Darmstadt, Germany). Sodium chloride, hydrochloric acid, sodium hydroxide, and Florisil were obtained from Carl Roth GmbH & Co. KG. (Karlsruhe, Germany). All chemicals were at least of analytical grade, and ultrapure water was used throughout the experiments.

### 2.2. Preparation of Pectin Solutions

Pectin solutions were prepared by dissolving 0.1 wt%, 0.25 wt%, 0.5 wt%, 1 wt%, or 2 wt% pectin in ultrapure water at 60 °C using an Ultraturrax T-25 digital dispersing system with a S25 KD—18 G dispersing tool (IKA^®^ Werke GmbH & Co. KG., Staufen, Germany) at a rotational speed of 10,000 rpm (tip speed 6.54 m/s) for 1 min. The pH of the solutions was adjusted to either 3, 4, or 5 by using 1 M HCl/NaOH at room temperature. Then, the solutions were left to equilibrate for at least 15 h, with gentle agitation using magnetic stirrer.

### 2.3. Zeta Potential and Hydrodynamic Diameter Measurement

The zeta potential and the average hydrodynamic diameter (in the following, reported as “hydrodynamic diameter”) of SBP in solution were determined with a particle analyzer Nanopartica SZ-100Z (Horiba Scientific, Kyoto, Japan). The zeta potential was obtained by electrophoretic light scattering using graphite electrodes. Pectin solutions (0.1 wt%) were prepared as described in Section 2.2, and the electrophoretic mobility was measured by the laser doppler electrophoresis technique. The zeta potential values were then calculated from the electrophoretic mobility using the Smoluchowski model. Measurements were conducted at least 10 times at a temperature of 25 °C.

For the determination of the hydrodynamic diameter, pectin solutions were diluted further to yield 8 diluted samples (0.005–0.1 wt%) and were given another 15 h equilibration time. The solutions were filtered using a 0.45 μm PTFE membrane (Carl Roth GmbH & Co. KG., Karlsruhe, Germany). Then, three measurements of 10 runs each with 90 s run time were conducted per sample. The refractive indices were set at *n* = 1.5470 for pectin and *n* = 1.333 for water for all measurements. The hydrodynamic diameter was obtained by plotting the measured z-average diameter versus pectin concentration and extrapolating it to zero pectin concentration (*R*^2^ = 0.87–0.96).

### 2.4. Pendant Drop Method

The interfacial activity and the adsorption behavior of SBP was determined by measuring the interfacial tension σ (IFT) using the pendant drop technique (OCA 15 LJ, DataPhysics Instruments GmbH, Filderstadt, Germany). The MCT oil was purified five times by mixing it with Florisil (Carl Roth GmbH + Co. KG., Karlsruhe, Germany) using the method described by Dopierala et al. [29]. A purified oil droplet with a volume of 12.5 µm was formed at the tip of the tensiometer capillary in the respective pectin solution (0.1 wt%) at 25 °C. Immediately after droplet formation, images of the droplet contour were taken for 15 h, and the interfacial tension was calculated by the device-specific software using the Young-Laplace equation:

(1) $$\gamma \left(\frac{1}{R_{1}}+\frac{1}{R_{2}}\right)=\Delta P=\Delta P_{0}-\Delta \rho \;gz$$

where *R*_1_ and *R*_2_ are the principal radii, ΔP is the Laplace pressure across the interface, and Δρ is the density difference between the droplet phase and the continuous phase. An overview of method and theory was given by Berry et al. [30]. As reference, measurements were performed with MCT oil and ultrapure water.

### 2.5. Quartz Crystal Microbalance with Dissipation Monitoring (QCM-D)

To characterize the adsorbed pectin mass and layer thickness, the adsorption film formed by SBP at solid-liquid interfaces was measured using quartz crystal microbalance with dissipation monitoring (QCM-D). QCM-D experiments were carried out using a qCell T instrument with qGraph software (3T GmbH & Co. KG., Tuttlingen, Germany). The sensor surface was coated hydrophobically to mimic an oil-water interface. To reuse the sensors, a cleaning procedure was performed and a fresh coating was applied after each measurement.

#### 2.5.1. Preparation of Self-Assembled Monolayers (SAMs)

To prepare the hydrophobically modified surface, clean gold-coated crystal sensors were exposed to 1 mM 1-dodecanethiol (≥98%) in absolute ethanol for 24 h using a coating unit (3T Analytik, Tuttlingen, Germany) [31,32,33]. Tissues soaked in ethanol were added to the headspace to prevent rapid evaporation of the thiol solution during the coating process. Afterwards, the gold sensors were thoroughly rinsed with ethanol and ultrapure water and dried using nitrogen gas. Subsequently, the crystal sensors were immediately used for the QCM-D experiments. For reuse, the crystal sensors were subjected to a cleaning procedure. For this purpose, they were cleaned with sulphuric acid-based piranha solution for 10 min and were then placed in a low pressure oxygen plasma cleaner Pico (Diener electronic GmbH & Co. KG., Ebhausen, Germany) for 10 min. The piranha solution was prepared by mixing hydrogen peroxide (30%) with concentrated sulphuric acid (98%) at a volume ratio of 1:3.

Contact angle measurements were used to prove a successful thiol coating on the gold surface. The contact angle of the coated sensors and ultrapure water was determined by droplet contour analysis and was ~90° (OCA 15 LJ, DataPhysics Instruments GmbH, Filderstadt, Germany).

#### 2.5.2. QCM-D Measurements

The QCM-D was operated at the fundamental frequency of the quartz crystals (~10 MHz). The variation in frequency (Δf) and dissipation (ΔD) due to SBP adsorption was used to calculate the adsorbed mass and the layer thickness on the interface. The experiments were started by pumping ultrapure water with adjusted pH (pH 3, 4, or 5, respectively) into the QCM-D chamber. Constant flow rates of 0.1 mL/min through the measurement chamber were maintained by a peristaltic pump (3T Analytik, Tuttlingen, Germany).

Pectin adsorption was measured after a stable baseline for frequency, and dissipation was established at 25 °C. Pectin solutions (0.1 wt% or 1 wt%, respectively; see Section 2.2) were filtered using 0.45 µm PVDF membranes and pumped into the chamber for 45 min. To remove loosely or unattached SBP after the adsorption step, the chamber was rinsed with ultrapure water (pH 3 or 5, respectively) for 20 min. The frequency and dissipation shifts depend on the adsorbed mass and the viscoelasticity of the pectin layer. The density of the respective SBP solutions was determined using a Gay-Lussac Pycnometer (Carl Roth GmbH & Co. KG., Karlsruhe, Germany). The viscosity value was chosen at 100 s^−1^ using rheometer Physica MCR 301 with double gap Couette geometry (Anton Paar GmbH, Graz, Austria) at 25 °C. The film density was assumed to be 1200 kg/m^3^, as for protein layers with trapped water [34]. Calculations of the data were performed with the device-specific software qGraph. Since pectins form a viscoelastic film (ΔD > 10% of Δf), the Voight model was used to calculate the layer thickness [34,35].

### 2.6. Emulsion Preparation and Characterization

Emulsions were prepared with MCT oil (1–30 wt%) as dispersed phase and pectin solutions (0.1–2 wt% pectin) as continuous aqueous phase. The pectin solutions were prepared as described in Section 2.2. Then, an emulsion premix was prepared by adding the oil phase while using an Ultraturrax T-25 digital at 15,000 rpm (9.82 m/s) for one minute. The emulsion premix was then transferred to a high-pressure homogenizer Microfluidizer^®^ MF 110 EH equipped with a Y-type interaction chamber (Microfluidics Corporation, Newton, MA, USA). The microchannel diameter of 75 µm was followed by an auxiliary processing module (APM) with a microchannel of 200 µm. The emulsions were homogenized at 400 bar, with an additional second pass at 800 bar. Droplet size distributions (DSD) of highly diluted emulsions were measured by static laser light scattering using a particle analyzer (HORIBA LA-950, Microtrac Retsch GmbH, Haan, Germany). The refractive indices were set at *n* = 1.333 for water and *n* = 1.4494 for MCT oil for all measurements. All measurements were conducted in triplicate at room temperature on the day of production and after storage for 30 days at 5 °C. The results are depicted as the cumulative volume distribution Q_3_ or the 90th percentile of the volumetric cumulative size distribution (d_90,3_) as characteristic values. Furthermore, microscope images were taken using an Eclipse LV100ND (Nikon GmbH, Düsseldorf, Germany) equipped with a DS-Fi1c camera in 20-fold magnification.

The viscosity of the investigated emulsions was determined with a Physica MCR 301 rotational Rheometer (Anton Paar, Graz, Austria). All measurements were conducted with a double gap geometry DG26.7 at 25 °C. Viscosity measurements were conducted at shear stress values ranging from 0.01 to 10 Pa. The stress was logarithmically increased, recording 10 measurement points for each decade.

### 2.7. Statistical Analysis

All measurements were conducted in triplicate if not stated otherwise. Statistical analysis, calculation of averages, and standard deviations were performed by using the software OriginPro 2019 (OriginLab Corp., Northampton, MA, USA). Significantly different mean values of variables (*p* < 0.05) were determined using Scheffé’s method.

## 3. Results and Discussion

### 3.1. Colloidal Properties of Pectins in Aqueous Solution

In order to evaluate the extent to which SBP molecules were unfolded, the hydrodynamic diameter of SBP was determined in aqueous solution at different pH values. Additionally, the zeta potential was measured to link the extent of unfolding to the amount of surface charge, which is also influenced by pH.

**Figure 1:**
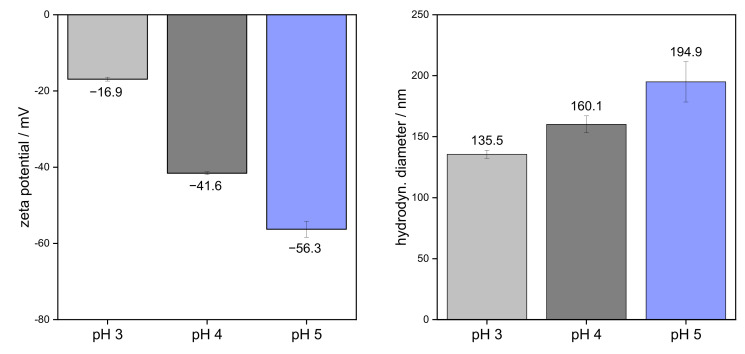
Zeta potential and hydrodynamic diameter of 0.1 wt% sugar beet pectin in aqueous solutions at different pH values.

Figure 1 shows the zeta potential values and hydrodynamic diameter of SBP in solution at pH 3, 4, and 5. SBP possessed the most negative zeta potential at pH 5. A high pH value leads to an increased dissociation of carboxyl groups and thus to an increased negative surface charge. Therefore, the dissociation of carboxyl groups and, consequently, the magnitude of zeta potential decreased with decreasing pH. This led to a strongly reduced zeta potential (pH 3: −16.9 ± 0.5 mV) at pH < 3.48, which is the pK_a_ of galacturonic acid [27]. This is in good agreement with Alba et al. [21], who showed a sharp decrease in the charge of okra pectins due to protonation of the carboxyl groups at pH values < 4. Moreover, Zeeb et al. [36] showed that for 0.5% pectin solutions, the lowest zeta potential and thus the highest surface charge occurred at pH 5 (zeta potential of approx. −32 mV). Li et al. [37] measured a zeta potential for 1.0 wt% pectin solutions after filtration through 0.45 µm membranes. In the study, a value of about −40 mV at pH 5 was obtained. However, significantly higher surface charges were obtained in our study (see Figure 1). The lower charges in the study of Zeeb et al. [36] and Li et al. [37] can be attributed to the use of buffer solution and preservatives, in which the positively charged sodium ions shield the negative charges. In addition, differences in the structure of pectins, e.g., different degrees of esterification, lead to different zeta potentials [24].

Before measuring the hydrodynamic diameter, all samples were filtered through 0.45 µm membranes. This could have separated very large pectins and aggregates before measurement. The polydispersity index of SBP indicated a narrow distribution and did not show significant differences between pH 3–5 (0.58–0.65 for 0.1 wt% pectin solutions). It can be shown that the measured hydrodynamic diameter increased in the order pH 5 > pH 4 > pH 3 (see Figure 1). This is due to the fact that intramolecular repulsions at pH > pK_a_ cause unfolding of the pectin chains due to electrostatic repulsions, whereas the conformation of SBP remains compact at pH 3 [25,26]. Therefore, SBP showed larger hydrodynamic diameter with increasing pH value. In another study, Siew, et al. [38] isolated SBP that was previously adsorbed onto limonene oil droplets. The calculated hydrodynamic radii of the SBP fractions in water were ~65 nm (corresponding diameter of 130 nm) at pH 4, which is below the measured diameter of 160 nm for SBP in this study. However, the difference can be attributed to different molecular structures.

### 3.2. Interfacial Properties of Pectins

The adsorption behavior at liquid-liquid and solid-liquid interfaces was investigated using both the pendant drop technique and quartz crystal microgravimetry. These methods are complementary models used to simulate the adsorption of emulsifier molecules on oil droplets in the emulsification process. The combination of the two methods allows a specific investigation of the adsorption and stabilization mechanism at different environmental conditions.

**Figure 2:**
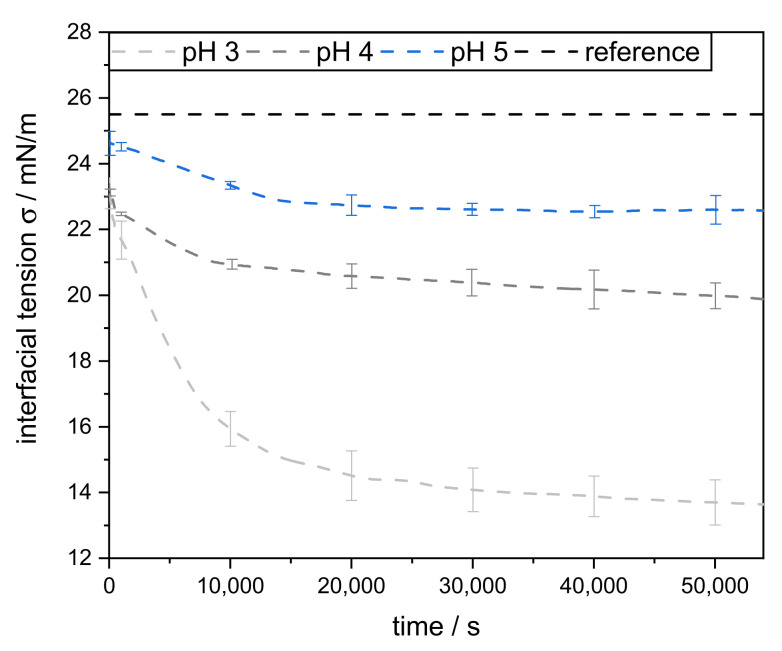
MCT oil-water interfacial tension, with 0.1 wt% sugar beet pectin in the aqueous phase, measured over the time at 25 °C. As a reference, the IFT (interfacial tension) was measured without the addition of sugar beet pectin.

In the pendant drop method, a liquid-liquid interface is considered, but the adsorption of the emulsifier molecules proceeds diffusively from a resting bulk phase. Measuring the decrease in interfacial tension over time can be used to obtain information about the adsorption process of SBP. The dynamic interfacial tension between MCT oil and 0.1 wt% pectin solutions was measured at pH 3, 4, and 5 for 15 h (Figure 2). At lower pH values, the IFT decreased significantly faster and more strongly (pH 3 > pH 4 > pH 5). This is in good agreement with Schmidt et al. [24], where the negative repulsion between interface and pectin molecules slowed down the adsorption kinetics of citrus pectin at pH 4 compared to pH 2.

Furthermore, for samples with pH 5, an equilibrium IFT was reached after about 8 h (~22.5 mN/m). At pH 3 and pH 4, the decrease in IFT continued even further after 15 h. However, the reduction was more pronounced at pH 3 (down to ~13.5 mN/m) than at pH 4 (~20.8 mN/m). This indicates that the largest amounts of SBP adsorbed onto the interface at pH 3 and probably allowed multilayer formation. This might be attributed to the more compact conformation at lower pH and less electrostatic repulsion.

In order to quantify the adsorbed mass and to obtain information about the layer thickness of the SBP films at the interface, experiments were performed using QCM-D. In contrast to the pendant drop method, in measurements using QCM-D, the emulsifier molecules adsorb to a hydrophobic solid surface, but they are transported to it in convective flow, which is closer to the actual conditions in the emulsification process.

In order to mimic an MCT oil-water interface, the sensor gold surface was modified by 1-dodecanethiol self-assembled monolayer. QCM-D measurements were performed at pH 3–5 with two different concentrations (0.1 wt% and 1.0 wt%). The decrease in frequency and increase in dissipation of pH 3 and 5 is shown in Figure S1. The adsorbed mass and layer thickness of all pectin solutions are shown in Table 1, alongside the corresponding density and viscosity used for the calculation.

The results support the assumption that minimized intra- and intermolecular repulsions at pH 3 lead to an increased SBP adsorption onto the hydrophobic surface. This is noticeable as a higher mass is adsorbed at pH 3 compared to pH 4 and pH 5 (Table 1). At 0.1 wt% SBP concentration, the adsorbed mass at pH 3 (524 ± 91 ng/cm^2^) was about twice that at pH 4 (257 ± 23 ng/cm^2^) and more than three times that at pH 5 (147 ± 39 ng/cm^2^). In addition, a significantly higher adsorbed mass was observed at 1 wt% SBP solutions than at 0.1 wt%. However, no significant differences were observed between pH 4 (388 ± 69 ng/cm^2^) and pH 5 (442 ± 148 ng/cm^2^). The same trend applies to the layer thickness. The layer thickness of SBP at the interface was also significantly greater with decreasing pH (1.2 ± 0.3 nm at pH 5 and 9.3 ± 0.7 nm at pH 3 for 0.1 wt% solutions) and increased with increasing concentration. Both the higher adsorbed mass and the greater layer thickness could be attributed to the compact conformation of pectin’s molecules, which was caused by a lesser degree of unfolding. As a result, polyelectrolytes such as SBP tend to adsorb to the surface in a more spherical conformation. This results in a greater layer thickness than in a more unfolded conformation, where SBP tends to adsorb flat onto the surface [39]. Whereas increasing the concentration at pH 3 led to a significant increase in adsorbed mass, the layer thickness did change slightly (0.1 wt% SBP: 9.3 nm, 1 wt% SBP: 11.6 nm). This suggests a compression of SBP at the interface. In addition to a lesser degree of unfolding at the interface, a higher film thickness could also be attributed to the additional formation of multilayers on the surface. This may be caused by hydrophobic attraction and hydrogen bonding, as well as by electrostatic complexation between the negatively charged galacturonic acid residues and the positively charged protein moiety [16].

**Figure 3:**
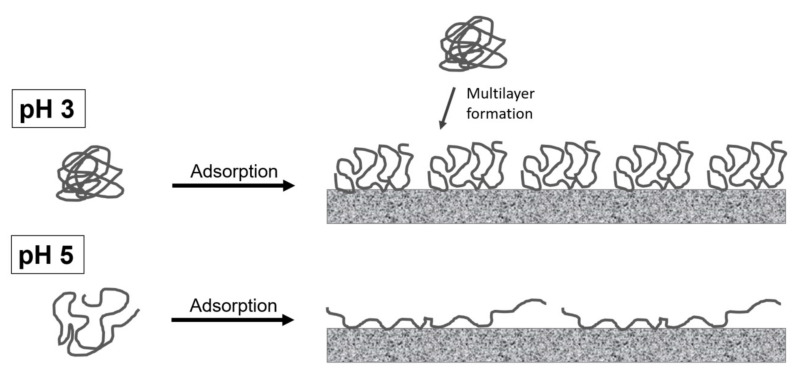
Schematic illustration of sugar beet pectin’s adsorption and conformation at hydrophobic surfaces at pH 3 (compact conformation) and pH 5 (unfolded conformation).

Overall, both the adsorption mechanisms and stabilization mechanisms at pH 4 were found to be intermediate between the characteristics of pH 3 and pH 5. However, the characteristics of SBP at pH 4 are more similar to the characteristics of SBP at pH 5 than at pH 3. SBP’s conformation at the interface, based on obtained results, is depicted schematically for pH 3 (compact conformation) and pH 5 (unfolded conformation) in Figure 3. On the one hand, more pectins can adsorb when the conformation is uncharged and compact. This leads to a denser and thicker adsorption layer, which can be attributed to an effective space occupancy and possible multilayer formation. This should be noticeable in effective steric stabilization, resulting in smaller emulsion droplets and more stable emulsions. On the other hand, SBP cover more interfacial area at pH 5 than at pH 3. This could be advantageous for low SBP concentrations. How this is reflected in the stabilization of oil droplets in emulsions will be considered in the following sections.

### 3.3. Characterization of Emulsions Prepared with Varying Pectin Concentration

As shown above, SBP adsorbed more rapidly and in greater quantity at lower pH-values, thus forming a thicker and more compact film at the interface. Therefore, when using high pressure homogenization, and sufficient pectin in the continuous phase, smaller droplets should be obtained at pH 3 than at pH 4 or pH 5. However, due to SBP’s compact structure, it is hypothesized that more pectins are required to sufficiently cover the interface and prevent coalescence. To verify this hypothesis, the pectin concentration in the continuous phase was varied using 0.1 wt%, 0.25 wt%, 0.5 wt%, 1.0 wt%, and 2.0 wt% as emulsifying agents.

**Figure 4:**
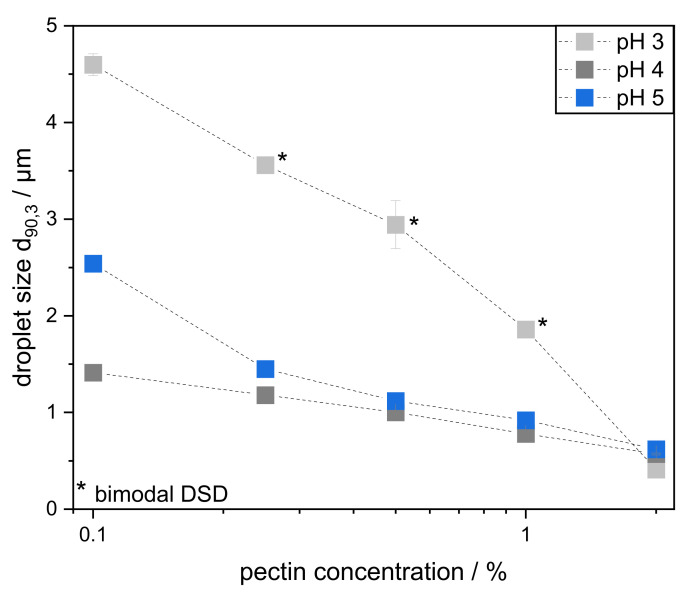
Characteristic droplet size d_90,3_ of emulsions prepared with 10 wt% MCT oil and varying pectin concentration of 0.1–2.0 wt% at different pH levels (3, 4, and 5). The emulsions were prepared with a high-pressure homogenizer at 400 bar and in a second pass at 800 bar. * Emulsion with bimodal droplet size distribution (DSD).

Depending on the pH, significant differences in droplet sizes occurred after high-pressure homogenization. The DSD of the emulsions are depicted in the supplementary material (Figure S2). In order to show the effect of pH and pectin concentration more clearly, Figure 4 shows the characteristic droplet size d_90,3_ plotted against the pectin concentration. The d_90,3_ was chosen for an easier comparison of the emulsions. Since there are a few bimodal DSDs (shown in Figure S2 and marked as * in Figure 4), the d_90,3_ is a more suitable choice for comparing the emulsions than the Sauter mean diameter. All d_90,3_ changed slightly in an unsignificant manner over a period of 30 days, so that long-term stability is given (Figure S4). Therefore, it can be assumed that most droplet coalescence occurred either during or immediately after the emulsification process. Droplets coalesced until the interface was small enough to be sufficiently stabilized by the pectins under the particular environmental conditions.

As expected, smaller droplet sizes correlated with higher pectin concentration for all emulsions. In general, only minor differences between pH 4 and 5 are visible at pectin concentrations above 0.5 wt%. At lower pectin concentrations, pH 4 resulted in smaller oil droplets than pH 5 due to a smaller hydrodynamic diameter, which resulted in a faster adsorption onto the surface and therefore a faster stabilization.

The droplet sizes of the emulsions at pH 3 differed in their dependence on pectin concentration: at low concentration, larger droplets, up to a factor of 3, were obtained at pH 3 than at pH 4 or 5. Consequently, the amount of pectin was deemed as insufficient to stabilize droplets effectively. The droplets coalesced until the interface reached a size that could be well stabilized. This is clearly reflected by the bimodalities of the DSD in Figure S2. A small fraction of the oil volume was stabilized immediately after droplet breakup. However, the entire oil-water interface could not be adequately covered, so coalescence occurred. This led to the appearance of larger droplets.

In contrast, when the pectin concentration was increased to 2.0 wt%, slightly smaller droplets were found in emulsions at pH 3 than at pH 4 or 5. This corresponds to findings by Nakauma et al. [12], who did not find significant differences in the DSD of emulsions prepared in a comparable manner but stabilized by slightly less SBP (1.5% SBP, 15% MCT oil, pH 3–5, high pressure homogenization at 500 bar).

Both observed phenomena are caused by the hydrodynamic size of the pectin molecules. If sufficient pectin is present, a smaller hydrodynamic diameter leads to faster adsorption at pH 3 and, consequently, to the stabilization of smaller droplets. This is in line with our results shown in Section 3.1 and Section 3.2 and with previous findings for citrus pectin [24]. However, if there is not enough pectin in the system to cover the interface adequately, a small hydrodynamic diameter is disadvantageous and leads to poorer stabilization. This is because, for the same mass, less interfacial area can be covered by more compact SBP. In addition, the absence of electrostatic repulsions between oil droplets could negatively affect emulsion stability at low pectin concentrations.

**Figure 5:**
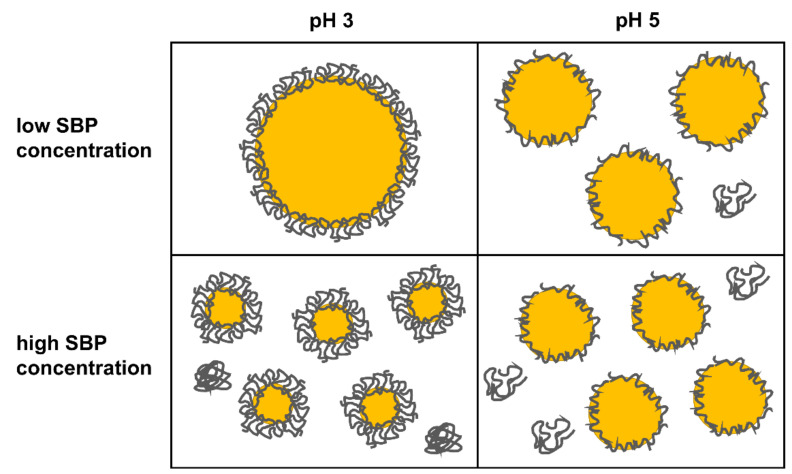
Schematic illustration of sugar beet pectin’s oil droplet stabilization at pH 3 (compact conformation) and pH 5 (unfolded conformation).

SBP’s conformation at the interface of oil droplets, based on obtained results, is depicted schematically for pH 3 (compact conformation) and pH 5 (unfolded conformation) in Figure 5.

### 3.4. Effect of Varying Oil Concentration

Emulsion-based food products contain various oil concentrations, depending on consumer demands, e.g., low-fat salad dressings typically include 10% oil—as used in the experiments above—whereas full-fat salad dressings comprise about 30% oil. At higher oil contents, droplet-droplet interactions occur more frequently, making emulsions more prone to coalescence.

So far, this study has shown that, above a certain pectin-to-oil ratio, the smallest droplets can be obtained at a pH of 3 (see Figure 4). In contrast, at low pectin-oil ratios, better results were obtained at pH 4 and pH 5 than at pH 3. However, this was only shown by varying the SBP concentration. In order to evaluate the effect of conformational differences on droplet stabilization at varying formulations, emulsions with different oil contents were prepared. Thereby, the SBP concentration in the continuous phase was kept constant at 0.5 wt%. In addition, the emulsions with 0.5 wt% SBP and 10 wt% oil, previously shown in Section 3.3, served as the reference sample. Thus, in addition to the 10 wt% emulsions, emulsions with reduced oil content (1 wt% and 5 wt%) and increased oil content (20 wt% and 30 wt%) were prepared.

**Figure 6:**
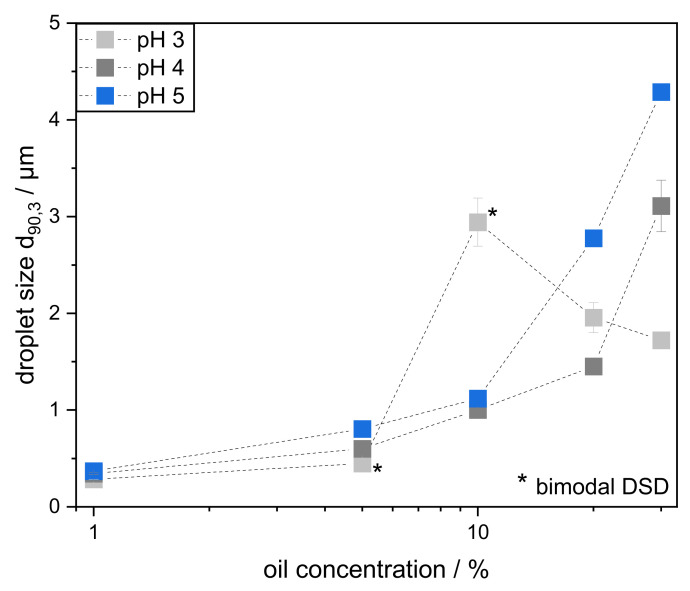
Characteristic droplet size d_90,3_ of emulsions prepared with 0.5 wt% pectin and varying oil content (1–30 wt%) at different pH levels (3, 4, and 5). The emulsions were prepared with a high-pressure homogenizer at 400 bar and in a second pass at 800 bar. * Emulsion with bimodal droplet size distribution (DSD).

On the one hand, reducing the oil content should result in sufficient pectin being present at all pH values, thus stabilizing the smallest droplets at pH 3. On the other hand, increasing the oil content should result in larger droplets, since there is more interface to cover. However, this effect should be more pronounced at pH 3 compared to pH 4 and 5, since less interface can be covered per molecule. In order to take a closer look at the influence of conformation at varying oil contents, the d_90,3_ of emulsions prepared with oil contents between 1–30 wt% are depicted in Figure 6. In addition, DSD of the emulsions is depicted in the supplementary material (Figure S3). None of the emulsions prepared showed signs of creaming during the observed storage period.

All emulsions prepared with 1 wt% MCT oil contained droplet sizes <0.4 µm. At the same time, all DSDs were narrow and monomodal. The use of smaller oil volumes reduced the collision frequency and minimized coalescence. Therefore, due to the high pectin-to-oil ratio, the DSD differed only slightly at different pH values, and it can be stated that very small droplet sizes can be achieved and stabilized with SBP at pH 3–5. The smallest droplets were obtained at pH 3 (d_90,3_: 0.28 µm), since SBP adsorbed faster and in higher quantities at lower pH, as seen previously in Section 3.2. So, the d_90,3_ of emulsions prepared with 1 wt% and 5 wt% oil decreased in the order pH 3 > pH 4 > pH 5. Again, a compact pectin conformation seems beneficial in stabilizing small amounts of oil.

As previously described, the interfacial coverage of emulsions prepared with 0.5 wt% pectin and 10 wt% oil was not sufficient, leading to bimodal DSD when pectin’s conformation was compact (pH 3). This was, however, not the case for SBP’s unfolded conformation (at pH 4 and 5). Since the droplet sizes at pH 4 were smaller than at pH 5 for all oil concentrations studied, electrostatic stabilization seems to play a minor role, and further unfolding of pectin molecules does not lead to any further improvement regarding droplet size. Instead, there seems to be an optimum between compactness for fast adsorption to the interface and unfolding for interfacial coverage. Due to relatively fast adsorption and sufficient interfacial occupancy, the optimum for the used SBP at the selected conditions is therefore in the region of pH 4.

For higher oil concentrations, the same behavior as with 10 wt% MCT oil (largest droplets at pH 3) was expected. However, emulsions prepared with 20 wt% and 30 wt% MCT oil showed different behavior: Here, despite leading to more compact conformation, lower pH values seem advantageous. With increasing the oil content to 20 wt%, the droplets at pH 3 became smaller than with 10 wt%. With 30 wt% MCT oil, the phenomenon is even more pronounced, resulting in smaller droplet sizes at pH 3 than at pH 4 and 5. However, unlike many other emulsions investigated, the samples with 20 wt% and 30 wt% MCT oil at pH 3 are not stable over time (see Figure S5).

To investigate this phenomenon, rheological measurements were carried out and micrographs were taken. The viscosity of the investigated samples was determined over the shear stress. Selected results are depicted in Figure S6 in the supplementary content. Emulsions with 10 wt% oil showed lower viscosity at pH 3 than at pH 4 and 5, which can be attributed to the bimodal distribution of droplet sizes and more compact SBP in the continuous phase. Furthermore, it can be shown that the viscosity of the emulsions increased with increasing oil content. This is particularly noticeable in the area of low shear stresses. The viscosity measurements at pH 3 indicate the formation of agglomerates in the emulsions with 20 wt% and, even more pronounced, with 30 wt% MCT oil. However, once the shear stress is high enough, the agglomerated droplets appear to have been separated from each other and flow as single droplets. Afterwards, the emulsions reach a Newtonian plateau.

Flocculation occurs only at high oil concentrations and at low pH due to low intermolecular repulsion. In contrast, at pH > pK_a_, as at pH 4 and 5, agglomeration does not occur, due to electrostatic repulsion between the pectin chains. This is reflected in the differences in zeta potential in Figure 1.

A flocculation or clustering of droplets leads to an increased viscosity, limits the mobility of the droplets, and thus leads to a lower collision frequency. This resulted in less coalescence after drop break-up and thus in smaller oil droplets than in emulsions without agglomeration (pH 4 and 5). However, since the droplets were not sufficiently covered with SBP, instabilities occurred during the storage period (Figure S5). Micrographs confirm flocculation at high oil concentrations and low pH values (Figure S7).

## 4. Conclusions

In the present study, we focused on the influence of the conformation of SBP on their interfacial and emulsifying properties. In order to investigate the conformation of SBP, the colloidal properties were determined at different pH levels (3, 4, and 5). Zeta potential increased with decreasing pH, from around −56 to −17 mV. Accordingly, hydrodynamic diameter reduced from 195 to 136 nm. Then, the adsorption and stabilization mechanisms were analyzed using a pendant drop tensiometer and QCM-D. It could be shown that the adsorption kinetics were faster with decreasing pH due to smaller hydrodynamic diameter. In addition, more compact SBP molecules adsorbed in greater quantity and formed a thicker interfacial layer than unfolded molecules.

The emulsifying properties were investigated by preparing emulsions with oil concentrations ranging from 1 to 30 wt% and pectin concentrations from 0.1 to 2 wt% and by monitoring the droplet size distributions on the day of production and after 30 days of storage. Overall, the present study showed that SBP led to emulsions that were highly stable over the entire storage time. The emulsions could be produced both at very low SBP concentrations (0.1 wt%) and at high oil concentrations (up to 30 wt%). However, the stability depends on SBP’s conformation. In the case of high pectin-to-oil ratios, the smallest droplets were stabilized at pH 3, when SBP molecules were compact and the positive effect of fast adsorption kinetics dominated droplet stabilization. In the case of low pectin-to-oil ratios, the negative effect of compact SBP molecules covering less interfacial area per molecule was dominant, meaning that small droplets could only be stabilized at a higher pH.

## Figures and Tables

**Table 1:** Density and viscosity of pectin solutions (0.1 wt% or 1 wt% SPB at pH 3, pH 4, or pH 5) as well as estimated adsorbed mass and layer thickness obtained with the Voight model.

	pH 3		pH 4		pH 5	
	0.1 wt%	1.0 wt%	0.1 wt%	1.0 wt%	0.1 wt%	1.0 wt%
Density (kg/m^3^)	1001	1003	1001	1003	1001	1003
Viscosity (mPa·s)	1.2	8.0	1.2	8.5	1.3	8.8
Adsorbed mass (ng/cm^2^)	524 ± 91	1462 ± 105	257 ± 23	388 ± 69	147 ± 39	442 ± 148
Layer thickness (nm)	9.3 ± 0.7	11.6 ± 0.9	2.1 ± 0.2	3.1 ± 0.6	1.2 ± 0.3	3.7 ± 0.8

## Data Availability

The datasets generated for this study are available on request to the corresponding author.

## Supplementary Materials

The following are available online at https://www.mdpi.com/article/10.3390/foods11020214/s1. Figure S1: Shifts in dissipation and frequency of QCM-D measurements due to SBP adsorption (left: 0.1 wt% SBP solu-tion, right: 1.0 wt% SBP solution. Figure S2: Volumetric oil droplet size distributions of emulsions (MCT oil in water) prepared with 0.1–2 wt% sugar beet pectin as emulsifying agent and 10 wt% MCT oil at different pH values. Emulsions were homogenized first at 400 bar and in a second pass at 800 bar. Figure S3: Volumetric oil droplet size distributions of emulsions (MCT oil in water) prepared with 0.5 wt% sugar pectin and 1–30 wt% MCT oil at different pH values. Emulsions were homogenized first at 400 bar and in a second pass at 800 bar. Figure S4: Characteristic droplet size d_90,3_ of emulsions prepared with 10 wt% MCT oil and varying pectin concentration 0.1–2.0 wt% at different pH (3, 4, and 5). Emulsions were homogenized first at 400 bar and in a second pass at 800 bar. The droplet sizes were measured on the day of preparation (0 d) and after 30 days of storage at 5 °C (30 d). Figure S5: Characteristic droplet size d_90,3_ of emulsions prepared with 0.5 wt% pectin and varying oil content (1–30 wt%) at different pH (3, 4, 5). Emulsions were homogenized first at 400 bar and in a second pass at 800 bar. The droplet sizes were measured on the day of preparation (0 d) and after 30 days of storage at 5 °C (30 d). Figure S6: Viscosity of emulsions prepared with 0.5 wt% pectin and varying oil content (10–30 wt%) at different pH (3, 4, 5). Emulsions were homogenized first at 400 bar and in a second pass at 800 bar. Figure S7: Micrographs of emulsions prepared with 0.5 wt% pectin at pH 3. Emulsions were prepared with 10 wt% (A, C) or 30 wt% MCT oil (B, D). Images were taken before (A, B) and after dilution (C, D) with ultrapure water (adjusted to pH 3).
