# Supplementary material for: Effect of Conformation of Sugar Beet Pectin on the Interfacial and Emulsifying Properties
Source: Foods. 2022 Jan 13;11(2):214. doi: 10.3390/foods11020214 (PMC8775170; doi:10.3390/foods11020214)
Supplement: Supplementary file 1 [file foods-11-00214-s001.zip › foods-1469899-supplementary.pdf]

foods

MDPI

Supplementary Content

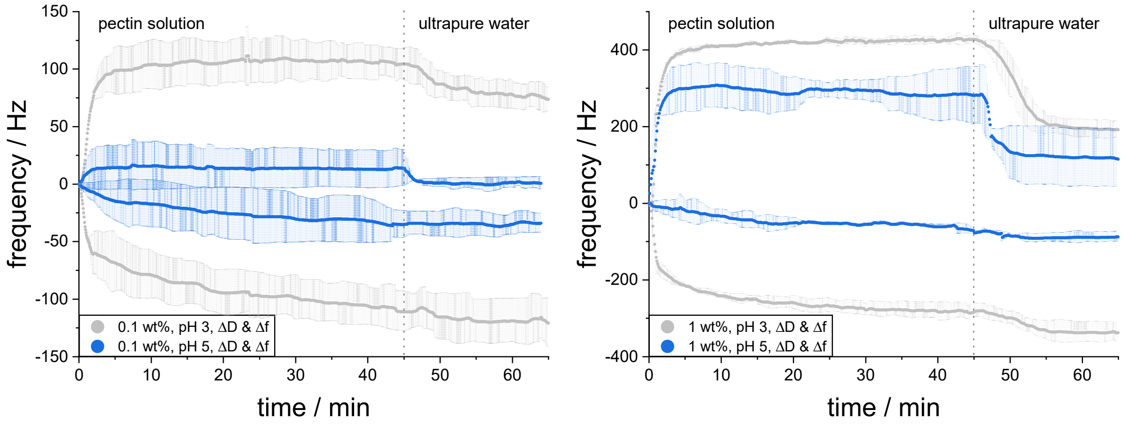

Figure S1. Shifts in dissipation and frequency of QCM-D measurements due to SBP adsorption (**left**: 0.1 wt% SBP solution, **right**: 1.0 wt% SBP solution).

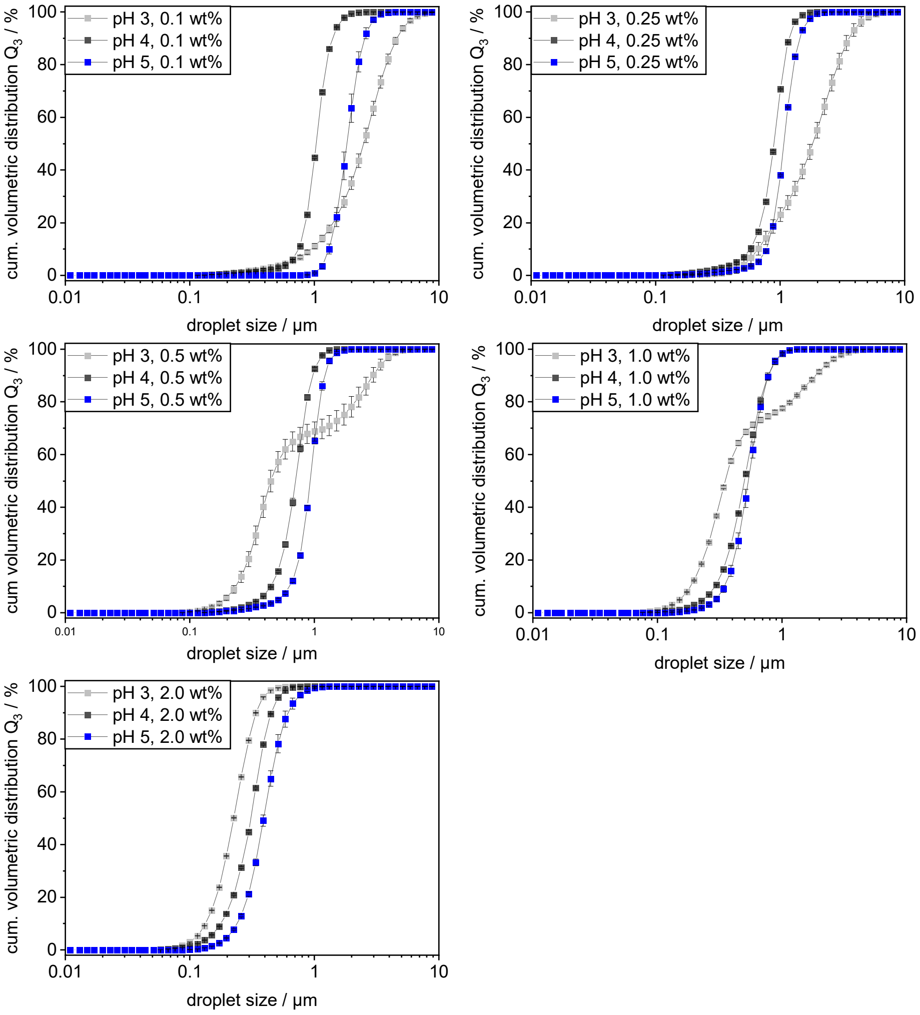

Figure S2. Volumetric oil droplet size distributions of emulsions (MCT oil in water) prepared with 0.1–2 wt% sugar beet pectin as emulsifying agent and 10 wt% MCT oil at different pH values. Emulsions were homogenized first at 400 bar and in a second pass at 800 bar.

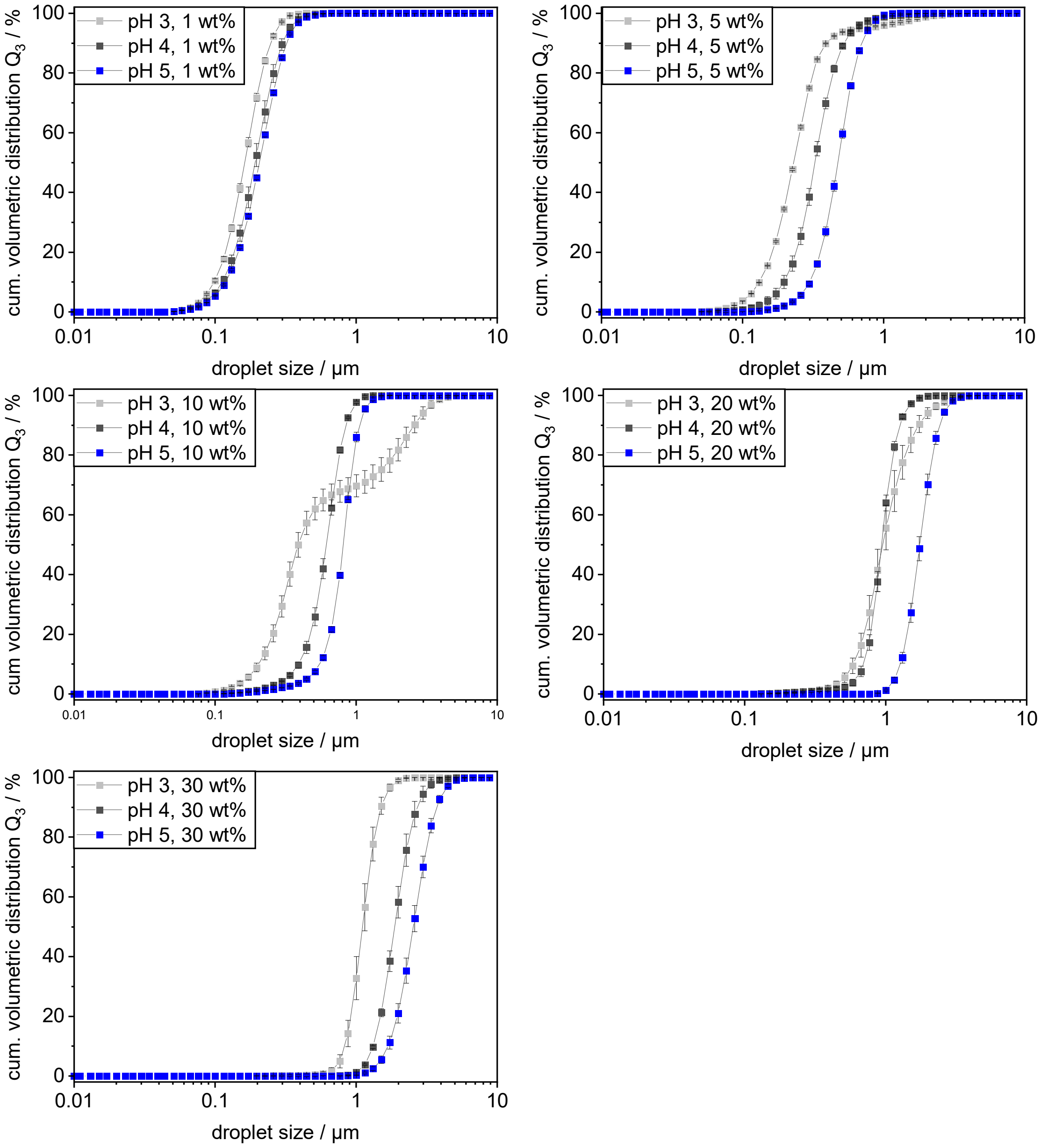

Figure S3. Volumetric oil droplet size distributions of emulsions (MCT oil in water) prepared with 0.5 wt% sugar pectin and 1–30 wt% MCT oil at different pH values. Emulsions were homogenized first at 400 bar and in a second pass at 800 bar.

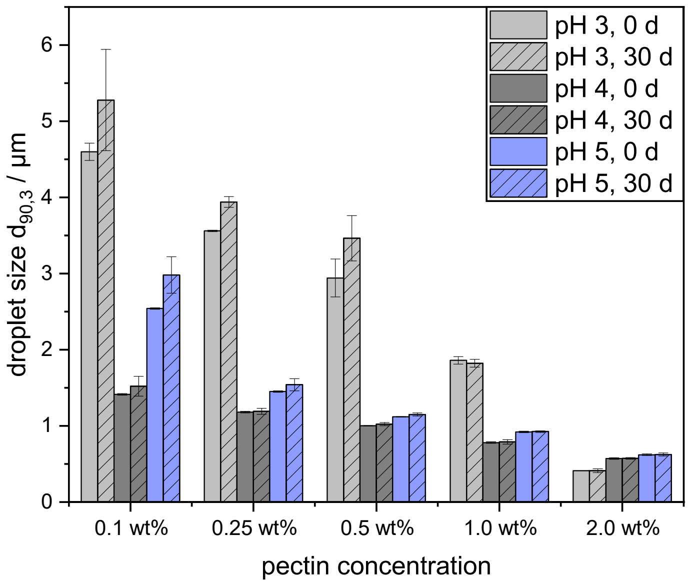

Figure S4. Characteristic droplet size $d_{90,3}$ of emulsions prepared with 10 wt% MCT oil and varying pectin concentration 0.1–2.0 wt% at different pH (3, 4, and 5). Emulsions were homogenized first at 400 bar and in a second pass at 800 bar. The droplet sizes were measured on the day of preparation (0 d) and after 30 days of storage at 5 °C (30 d).

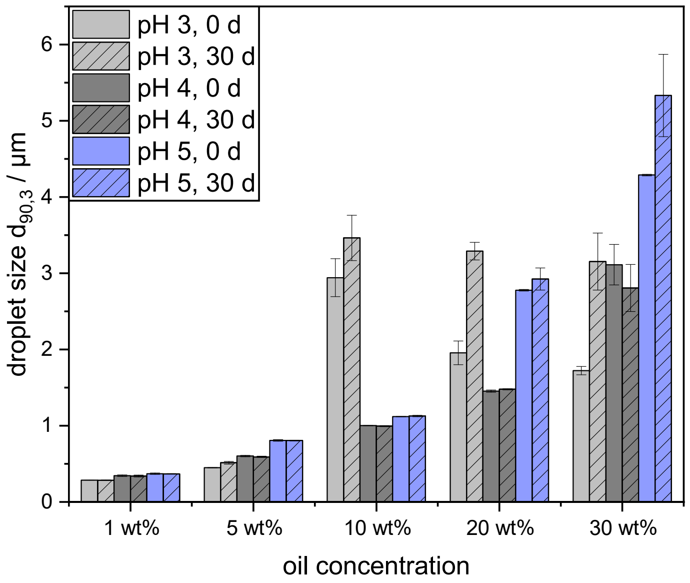

Figure S5. Characteristic droplet size $d_{90,3}$ of emulsions prepared with 0.5 wt% pectin and varying oil content (1–30 wt%) at different pH (3, 4, 5). Emulsions were homogenized first at 400 bar and in a second pass at 800 bar. The droplet sizes were measured on the day of preparation (0 d) and after 30 days of storage at 5 °C (30 d).

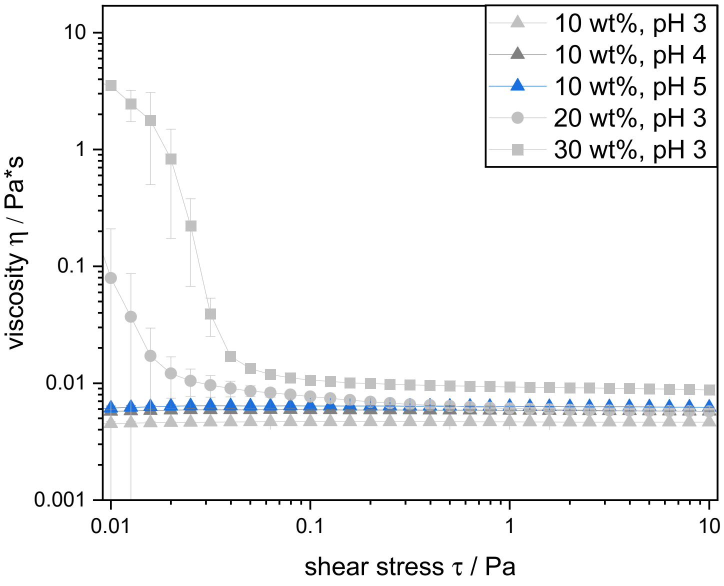

Figure S6. Viscosity of emulsions prepared with 0.5 wt% pectin and varying oil content (10–30 wt%) at different pH (3, 4, 5). Emulsions were homogenized first at 400 bar and in a second pass at 800 bar. Measurements were conducted at 25 °C with a double gap DG26.7 geometry. Images from emulsions at pH 3 were taken before and after dilution with ultrapure water, which was adjusted to pH 3. As already seen in the DSD in Figure S3 and Figure 6, smaller droplets could be observed for emulsions prepared with 30 wt% oil (Figure S7B,D) than with 10 wt% oil (Figure S7A,C). In addition, the bimodality is evident in emulsions prepared with 10 wt% oil. In contrast to the emulsion samples with 10 wt% oil, large cluster consisting of small droplets were visible in the undiluted samples with 30 wt% oil. Moreover, micrographs of the diluted samples showed that droplet agglomeration is reversible. Therefore, individual droplets and not agglomerates were recorded in the DSD measurement.

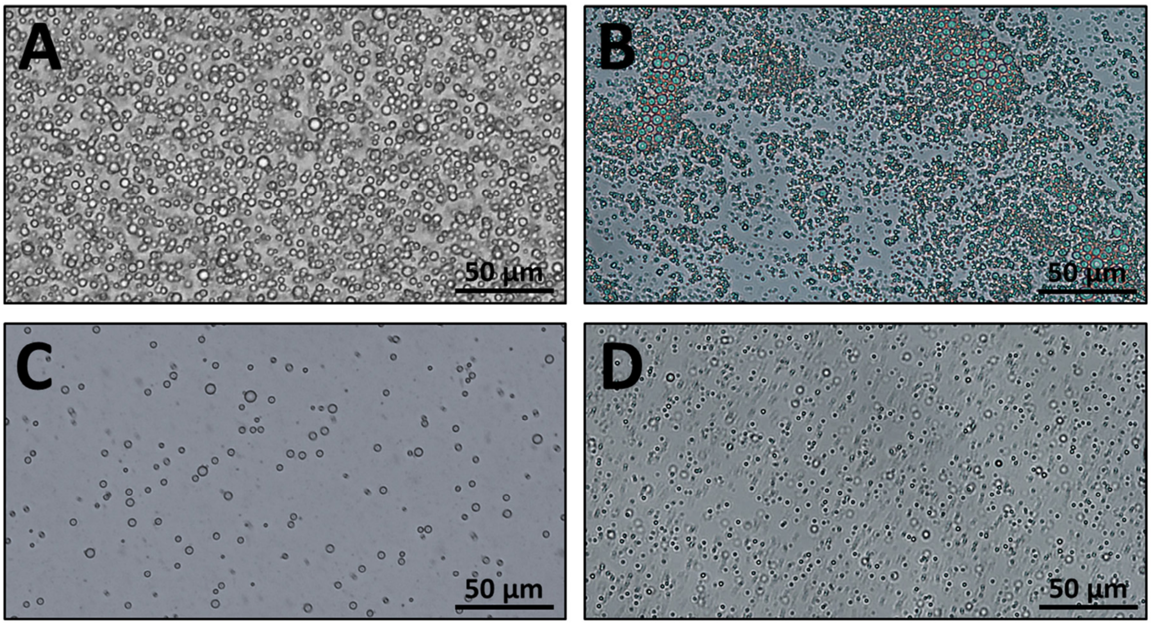

Figure S7. Micrographs of emulsions prepared with 0.5 wt% pectin at pH 3. Emulsions were prepared with 10 wt% (A, C) or 30 wt% MCT oil (B, D). Images were taken before (A, B) and after dilution (C, D) with ultrapure water (adjusted to pH 3).
